# The Practice of Weight Loss in Combat Sports Athletes: A Systematic Review

**DOI:** 10.3390/nu16071050

**Published:** 2024-04-03

**Authors:** Yuming Zhong, Yuou Song, Guilherme Giannini Artioli, Thomas I. Gee, Duncan N. French, Hang Zheng, Mengde Lyu, Yongming Li

**Affiliations:** 1 School of Athletic Performance, Shanghai University of Sport, Changhai Road 399, Yangpu District, Shanghai 200438, China; 2221111051@sus.edu.cn (Y.Z.); 2321852033@sus.edu.cn (Y.S.); 2311811001@sus.edu.cn (H.Z.); mengdelyu@sus.edu.cn (M.L.); 2 Department of Life Sciences, Manchester Metropolitan University, Manchester M15 6BH, UK; g.giannini.artioli@mmu.ac.uk; 3 School of Sport and Exercise Science, University of Lincoln, Lincoln LN6 7TS, UK; tgee@lincoln.ac.uk; 4 Ultimate Fighting Championship Performance Institute, Las Vegas, NV 89118, USA; dfrench@ufc.com; 5 China Institute of Sport Science, Beijing 100061, China

**Keywords:** weight cutting, weight management, weight cycling, RWL, body mass

## Abstract

The aim of this systematic review is to comprehensively assess the weight loss (WL) practices in different combat sports (CS). The review protocol was preregistered with PROSPERO [CRD42023487196]. Three databases were searched (Web of Science, EBSCOhost, and PubMed) until 8 December 2023. Eligible studies had to meet five criteria: they must have been (a) written in English, (b) published in a peer-reviewed journal, (c) used a survey design to investigate the WL practices of CS athletes, and (d) reported the WL methods used by athletes using a five-point scale. Twenty-six studies (3994 participants from 14 CS) were included. This review found that (1) WL is highly prevalent in CS athletes; (2) many CS athletes started losing weight for competition as teenagers two to three times a year; (3) CS athletes usually lose <5% body weight in 7–14 days before competition; (4) increasing exercise and gradually dieting are the most commonly used WL methods; and (5) the influence of scientific practitioners on athletes is negligible. The habitual practices of CS athletes may be relatively harmless, but in some special cases, CS athletes also perform extreme WL practices. Scientific practitioners have little influence on their WL practices, which may form a vicious cycle of non-qualified influence.

## 1. Introduction

Combat sports (CS) have been rapidly growing in popularity over the past two decades. In the 2020 Olympic Games, there were six CS, accounting for about 22% of the gold medals. In professional sports, CS has attracted millions of spectators, especially in the cases of professional boxing and mixed martial arts [1]. The rapid development of CS has led to more athletes participating in combat competitions and more competitions for athletes each year.

In CS, in order to prevent unfair competition caused by differences in size and strength, athletes are grouped into weight classes. Athletes’ weights are verified at the official “weigh-in”, and those who exceed their class limit may be disqualified. Therefore, choosing the “right” weight class and manipulating weight accordingly is important for all CS athletes. To gain a competitive advantage, CS athletes (excluding those in an unlimited weight class) often use a variety of methods to lose weight rapidly before competitions, many of which can pose a health risk [2,3,4,5,6,7]. Many past studies have found that athletes most often achieve their target weight by restricting their food/nutrient intake (e.g., not eating all day) [2,3,4,5,6,7]. Given that unhealthy weight loss (WL) practices endanger the health of athletes [1,8,9,10,11] and increase the risk of competing, it is important to investigate the WL practices used in the real world.

A number of surveys were conducted to understand and reveal the details of the WL practices among CS athletes. These studies were conducted in different countries (e.g., Brazil, France, and Australia) [6,12,13] and considered all competitive levels (e.g., high school, university, and elite level) [14,15,16,17], and different CS modalities (e.g., judo, taekwondo, and boxing) [18,19,20]. Although there is a large number of existing studies on this topic, those conducted before 2010 provide limited comparative data (usually only prevalence and magnitude of WL can be compared across studies) because they are not based on the same questionnaire or similar ones [14,21,22,23,24,25,26,27,28,29,30,31,32]. In 2010, Artioli et al. established a rapid WL questionnaire (RWLQ) and tested its reliability and validity [33]. Since then, most of the studies surveying the WL practices of CS athletes have adapted this questionnaire [2,3,4,5,6,7,12,13,15,16,17,18,19,20,34,35,36,37,38,39,40,41,42,43,44,45,46]. Although some studies have further explored other fields (e.g., physiological symptoms, psychological symptoms, perceived influence, and injury rate) [36,37,41], the original content of the survey is always applicable. Specifically, each study involved CS athletes’ demographic information, competition performance information, weight history, WL methods, and person(s) who influence their WL practice(s).

In the real world, the WL practices of CS athletes are affected by many factors, including athletes’ national culture, competitive level, and combat modality; the people around them; and their personal habits [1,6,12,15,20,47]. Therefore, there will be some differences in the results of studies surveying CS athletes across differing backgrounds. For example, the WL practices among jiu-jitsu athletes have been shown to differ from those of MMA athletes [13,45]; likewise, the WL practices of elite athletes are different from those of non-elite athletes [6,20,45]. These differences limit generalization and the ability to draw conclusions from different studies [12,20,42,48]. In fact, due to the different sample backgrounds, differences in the WL practices reported are inevitable. Further analysis of the commonalities and differences in the practice of athletes from different backgrounds is necessary for understanding and improving the practice of WL in CS. Given the importance of WL in CS and the rich literature available on the practice of WL in CS, it would be valuable to collate and compare data across sports.

Therefore, the purpose of this systematic review was to comprehensively assess the evidence of previous investigations into WL practices in different CS to further understand the patterns of WL practices among CS athletes with different backgrounds and determine whether the WL practices of athletes are consistent with existing research and evidence-based guidelines. This systematic review provides more comprehensive evidence and potential research directions for the study of this topic, and it also provides a basis for further promoting the scientific WL practice of combat athletes.

## 2. Materials and Methods

### 2.1. Registration of Systematic Review Protocol

**Figure 1:**
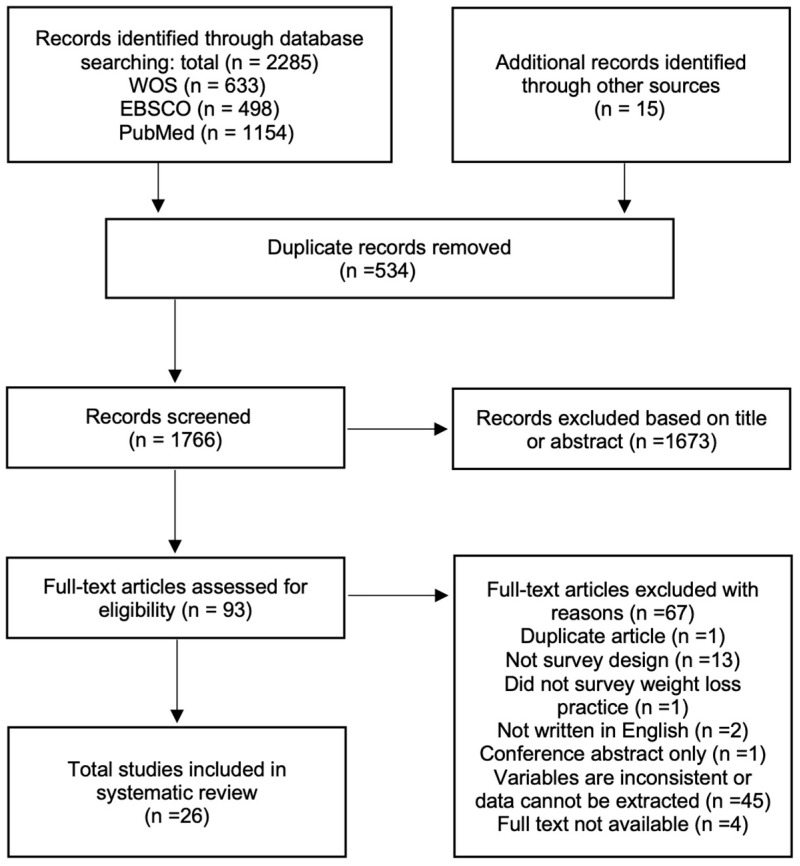
Preferred Reporting Items for Systematic Reviews and Meta–Analyses Protocol (PRISMA–P) flowchart illustrating the inclusion and exclusion criteria used in the systematic review.

To ensure transparent and complete reporting, this review followed the Preferred Reporting Items for Systematic reviews and Meta-Analysis guidelines [49] (see Figure 1). The study protocol was preregistered on the PROSPERO International Prospective Register of Systematic Reviews [CRD42023487196].

### 2.2. Information Sources and Search Strategy

Searches for studies were conducted by the lead author [YMZ] from 5 December 2023 to 8 December 2023 using three electronic databases considered suitable for systematic reviews (Web of Science, EBSCOhost, and PubMed) and an academic application (ResearchGate) [50]. The search string used in Web of Science was as follows: (Title = (“weight loss” OR “weight reduc*” OR “weight manage*” OR “weight control” OR “weight cut*” OR “body mass loss” OR “body mass reduc*” OR “body mass manage*” OR “body mass control” OR “body mass cut*”)) AND Topic Search = (“sport*” OR “athlet*”); the search string used in EBSCOhost was as follows: Title (“weight loss” OR “weight reduc*” OR “weight manage*” OR “weight control” OR “weight cut*” OR “body mass loss” OR “body mass reduc*” OR “body mass manage*” OR “body mass control” OR “body mass cut*”) AND Title (“practice*” OR “strateg*” OR “method*” OR “use*” OR “habit*” OR “Prevalence” OR “Magnitude”) AND All Text (“sport*” OR “athlet*”); the search string used in PubMed was as follows: (“weight loss” [Title] OR “weight reduc*” [Title] OR “weight manage*” [Title] OR “weight control” [Title] OR “weight cut*” [Title] OR “body mass loss” [Title] OR “body mass reduc*” [Title] OR “body mass manage*” [Title] OR “body mass control” [Title] OR “body mass cut*” [Title]) AND (“sport*” OR “athlet*”). The search terms used in ResearchGate were as follows: weight loss, weight reduction, weight management, weight control, weight cut, body mass loss, body mass reduction, body mass management, body mass control, and body mass cut. The reference lists of the selected studies were searched for additional suitable studies.

### 2.3. Eligibility Criteria

Studies were eligible if they met the following inclusion criteria: (a) written in English, (b) published in a peer-reviewed journal, (c) used a survey design to investigate the WL practices of CS athletes, and (d) reported WL methods used by athletes using a 5-point scale.

### 2.4. Study Selection

Duplicate references were first removed using the EndNote reference manager (version 20; Clarivate Analytics, Philadelphia, PA, USA). Following the removal of duplicates, the titles and abstracts of the identified articles were reviewed by two authors (YMZ and YOS) for initial eligibility. The authors were blinded to avoid bias during this process. Thereafter, the authors (YMZ and YOS) independently screened the full texts to determine inclusion eligibility based on the inclusion and exclusion criteria. Any disagreements between the reviewers’ decisions were discussed and, if unresolved, settled by a third reviewer (YML).

### 2.5. Data Collection Process and Data Items

From the included studies, the following data were extracted by two independent authors (YMZ and YOS) and checked by a third author (YML): (1) study information, (2) study appraisal rating, (3) published year, (4) sample size, (5) country, (6) sport, (7) gender, (8) competitive level, (9) age, (10) body weight (BW), (11) height, (12) age at start of training, (13) age at start of competing, (14) prevalence of WL, (15) frequency of WL in past 12 months, (16) highest WL (kg and BW%), (17) habitual WL (kg and BW%), (18) typical BW regain in a week after competition (kg and BW%), (19) age when starting WL, (20) number of days over which weight is usually lost, (21) methods used for WL, and (22) persons who influence their WL practice. If the data entered by two authors were inconsistent, the two authors discussed and determined the data. If data were missing or unextractable, an attempt was made to contact the study investigators to obtain the necessary data. If the study authors were unresponsive or unreachable, three authors reviewed the manuscript and confirmed whether not attainable (—) should be used. For this systematic review, the weight loss method score (WLMS) and person influence score (PIS) were further calculated according to Artioli’s WL score calculation rule [33]. The WLMS is calculated using the formula: (percentage of sample who select “Always” *3 + percentage of sample who select “Sometimes” *2 + percentage of sample who select “Almost never” *1 + percentage of sample who select “Don’t use anymore” *0.5) *100. The PIS is calculated using the formula: (percentage of sample who select “Not influential” *1 + percentage of sample who select “A little influential” *2 + percentage of sample who select “Unsure” *3 + percentage of sample who select “Somewhat influential” *4 + percentage of sample who select “Very influential” *5) *100.

### 2.6. Study Risk of Bias Assessment

The appraisal tool for cross-sectional studies (AXIS) was used to assess the quality of the studies included in this systematic review. AXIS is a technical quality assessment tool that critically evaluates cross-sectional studies and consists of 20 items. Five categories were evaluated: objectives, methods, results, discussion, and other (ethics and sponsorship). Each question is graded as either: yes, no, or don’t know. Two authors (YMZ and YOS) independently evaluated the studies. Any disagreements between the reviewers’ decisions were discussed and, if unresolved, settled by a third reviewer (YML).

## 3. Results

### 3.1. Study Selection

The search strategy and selection process resulted in 26 full-text studies being eligible for inclusion in the review (see Figure 1).

### 3.2. Study and Participant Characteristics

The included studies were published between 2010 and 2023 (Table 1). Fourteen CS modalities were represented (Judo *n* = 9, Taekwondo *n* = 7, MMA *n* = 6, Wrestling *n* = 5, Boxing *n* = 5, Karate *n* = 4, Sambo *n* = 3, Jiu-jitsu *n* = 3, Kickboxing *n* = 2, Muay Thai *n* = 2, Sanda *n* = 1, Silat *n* = 1, Viet Vo Dao *n* = 1, Yoseikan Budo *n* = 1), encompassing data from a total of 3994 participants. Six of the included studies covered multiple CS, and two included a small number of strength sports athletes (which did not affect the overall results). Of the included studies, seven included male athletes only, one included female athletes only, and the others included both male and female athletes. Regarding the competitive levels, six studies included international-level athletes only, one included national-level athletes only, and the others did not limit the competitive level. Fourteen studies did not explicitly state the proportion of participants with different competitive levels but simply qualitatively described the levels covered.

### 3.3. Study Risk of Bias Assessment

This study evaluated the collected studies using AXIS (Table 2). Of the 20 questions, most of the answers to questions 3 (25 studies), 7 (25 studies), and 14 (26 studies) were N, most of the answers to questions 5 (18 studies) and 13 (26 studies) were D, and most of the answers to the other questions were Y.

### 3.4. Prevalence of WL

All studies reported the prevalence of WL (Table 1). The prevalence of WL was high across the combat sports (66–100%). Twenty-three studies reported that prevalence of WL was more than 80%. Three exceptions were found in visually impaired judo (67%), jiu-jitsu (59%), and multiple CS (66%).

### 3.5. Weight History

Twenty studies reported full or partial results from this section’s topic (Table 3). Only participants who reported that they lose weight before competition answered the following questions and sections.

Seventeen studies reported the age at start of WL, ranging from 12.5 to 21 years, of which ten studies reported that some athletes begin partaking in WL activities at less than 18 years old. Thirteen studies reported the times of WL in the past year, ranging from 1.3 to 4.7 years, of which eleven studies reported 2.0–3.9 times.

Twenty-two studies reported the habitual magnitude of WL in kg (range: 1.3–10 kg), and twenty-one studies reported it as a percentage of body weight (range: 1.8–13% of BW), of which only three modalities from nine studies (MMA *n* = 5, Sambo *n* = 3, and Muay Thai *n* = 1) reported that athletes lost more than five percent of BW. Seventeen studies reported the highest magnitude of WL in kg (range: 2.7–13.9 kg), and fifteen studies reported it as a percentage of body weight (range: 4.7–17.5% of BW), of which fourteen studies reported that athletes lost more than five percent of BW.

Seventeen studies reported the number of days allocated to WL, ranging from 7 to 29.1 days, of which 15 studies reported more than 10.0 days.

### 3.6. WL Methods Used

All studies reported the WL methods used by participants on a five-point scale (Table 4). Increasing exercise (218/300) and gradually dieting (208/300) were the most commonly used WL methods for CS athletes. The methods with a score of more than 200 were increasing exercise, gradually dieting, and restricting fluid ingestion. Most of the methods used had a score between 100 and 200. The methods with a score of less than 100 are the use of rubber/plastic layers without exercising, laxatives, diuretics, diet pills, and vomiting.

### 3.7. Person Who Influenced Their WL Practice

Seventeen studies reported the person who influenced the athlete’s WL practice on a five-point scale. Coaches (368) had the most influence on athletes’ WL practices, while doctors/physicians (192/500) had a negligible influence (Table 5).

## 4. Discussion

This systematic review investigated the WL practices of CS athletes with different backgrounds. Based on the results from twenty-six studies, we found that (1) WL is highly prevalent (66–100%) in CS athletes, despite the differences in sample backgrounds across the studies; (2) many CS athletes started losing weight for competition as teenagers two to three times a year; (3) CS athletes usually lose <5% of their BW in 7–14 days before competition, and Sambo, MMA, and Muay Thai athletes lose more weight; (4) increasing exercise and gradually dieting are the most commonly used WL methods for combat athletes; (5) the more harmful the WL method is, the lower the score is, but the phenomenon of using harmful methods is still widespread; (6) the influence of scientific practitioners on athletes is negligible. This review found that WL is highly prevalent in CS athletes, but their habitual practice (except for Sambo, MMA, and Muay Thai athletes) may not commonly be significantly harmful. Nevertheless, extreme WL practice by CS athletes in special situations (e.g., experiencing a holiday) is a cause for concern, and the perceived influence of scientific practitioners is minimal.

### 4.1. Prevalence of WL

The results obtained showed that despite the differences in the backgrounds of the samples of the included studies, the prevalence of pre-competition WL in CS athletes was high, and only three studies reported a prevalence of WL less than 80% [3,37,45]. Further analysis found that there were some particularities in the sample backgrounds of three studies (namely studies on visually impaired athletes, athletes who started CS training [25.8 years] and competing [26.7 years] very late, and non-elite athletes). Previous research has shown that athletes with a higher competitive level or more competitive experience show a higher prevalence of WL and more aggressive WL behaviors [6,20,45,55]. The results acquired show that country and gender were not the main factors affecting the prevalence of WL, while sports and competitive level are. Specifically, more popular sports in the world usually involve more athletes, and the relative competitive standard will be higher. Similarly, the higher the level of competition within each sport, the more intense the competition among athletes. Therefore, athletes need to gain more advantages (e.g., height, arm span, strength) through WL to increase their possibility of winning competitions. While losing weight may not lead to intrinsic performance improvements [56,57,58,59] or a higher probability of winning [60,61,62], they believe it will lead to an advantage [63].

### 4.2. Weight History

According to the mean values of the included studies, the age at the start of WL of the CS athletes was from 12.5 to 21 years old, the frequency of WL in the past year was from 1.3 to 4.7 times, the habitual magnitude of WL was from 1.3 to 10 kg (1.8 to 13% of BW), the highest amount of weight lost was from 2.7 to 13.9 kg (4.7 to 17.5% of BW), and the number of days allocated to WL was from 7 to 29.1 days. Although the ranges of the overall results are large, most of the studies show similar results, and only a few studies have significantly different results.

*Age starting WL:* The results showed that the mean age range for starting WL among the CS athletes is very wide (from 12.5 to 21 years old). More than half of the studies (10/17) reported that the mean age at the start of WL of the participants was less than 18 years old, of which two studies reported that the mean age at the start of WL was 12 years old. In this review, we found that the age at the start of WL is related to the age at one begins competing, that is, athletes begin to lose weight after they start to participate in competitions. Further, all studies that reported both the age at the start of WL and the age at which one starts entering competitions reported that the age at the start of WL was higher than the age at which one started competing [3,4,6,7,13,16,18,19,20,34,39], indicating that most CS athletes do not perform pre-competition WL at their first competition but that they do perform WL after several competitions or after several years. At present, it is not clear why athletes do not lose weight at the beginning of their competitive careers, and after a period of time, they begin to lose weight before competitions. When examining the specific age range reported in each study rather than the mean value, it was found that most studies reported that some athletes started to lose weight very early [4,16,18,20,34,39]. For example, studies have reported that four-year-old judo athletes and five-year-old wrestlers lose weight in order to compete [34,64]. It is a worrying phenomenon that most athletes begin to lose weight from an early age. Because adolescence is the main period for the growth and development of athletes, starting WL at an earlier age will inevitably have some negative impact on the growth and development of athletes [65,66,67,68] and potentially lead to more extreme WL behavior in the future [55].

*Annual WL times*: For the mean number of pre-competition WL activities in the past 12 months, most of the studies reported two or three times. Six studies reported both the number of competitions participated and times of WL of participants in the past year [12,13,16,19,52,53], and five found that the mean times of WL was lower than the mean number of competitions participated in [12,16,19]. This seems to indicate that most athletes do not lose weight before every competition, especially those with a lot of competitions; rather, according to the specific situation (such as the importance of the competition, the interval between competitions, and their physical condition), athletes decide whether to lose weight. For example, the mean number of competitions participated in among Australia’s Olympic CS athletes was 7.1 times, including both the Olympic Games and national competitions [12]. If WL is carried out every time, it will likely affect the physical and mental health and competitive performance of athletes [1]. In addition, athletes will also decide how much weight to lose according to the specific circumstances. All studies showed that the maximum WL of athletes is greater than the habitual WL [3,6,7,12,13,16,18,19,20,34,39,51,52,53,54], and two studies showed a very large difference in this regard (10% vs. 4% of BW) [3,7].

*Magnitude of WL:* This review found that the magnitude range of WL in the included studies was very wide (habitual WL was from 1.3 to 10 kg [1.8 to 13% of BW]; highest WL was from 2.7 to 13.9 kg [4.7 to 17.5% of BW]). A recently published meta-analysis recommended that the WL of CS athletes should not exceed 5% of their BW [58]. Nearly two thirds (13/21) of the studies reported that athletes usually lose less than 5% of their BW on average; almost all (14/15) of the studies reported that athletes lost more than 5% of their maximum BW, and even seven studies reported that athletes’ WL exceeded 10% of their BW. Further analysis found that there is a clear sports specificity in the magnitude of WL in CS. All studies that reported habitual WL to be more than 5% of BW were studies surveying Sambo, MMA, and Muay Thai athletes, and the magnitude of WL values reported by studies surveying MMA athletes were the highest among all the included studies. The likely reason for this is that MMA weigh-in times are typically 24–36 h before competition, whereas those of Olympic combat sports are typically around <2 h pre-competition, and MMA includes one-bout competitions. Therefore, the WL values of MMA athletes were far greater than those of other CS athletes. Except for the studies of Sambo, MMA, and Muay Thai athletes, all studies reported that the athletes’ habitual WL was less than 5%, which is in line with the above research recommendations. Although previous studies addressing the effects of RWL on physiological parameters have produced conflicting data, demonstrating negative [69,70,71], positive [72], and negligible [72,73] effects, some systematic reviews and meta-analyses have shown no or negative effects of RWL on athletic performance [56,57,58,74].

*Days allocated to WL before competition:* Almost all studies that provided mean days (13/17) reported that the mean number of days allocated to WL before competition was between 7 and 14 days [3,4,6,7,12,15,16,18,19,20,34,39,44,52,54]. However, it should be pointed out that there are some limitations in using the mean value to show this metric, because 7 is already close to the minimum value of WL days, and the maximum value may be 30–60 days or even more, which is misleading to some extent. Therefore, some studies reported the percentage of different days range, which showed that the percentage of athletes who began to lose weight 0–7 days before competition was generally higher than the percentages of athletes who begin WL activities 8–14 days and >15 days before competition [20,37,42,45,46]. This may be because WL will affect the mood of athletes [44,56], and most athletes generally lose weight within an acceptable range (<5% of BW). Therefore, CS athletes prefer to complete their WL activities in a shorter period of time.

### 4.3. WL Methods Used

The results showed that regardless of the research background, increasing exercise (218) or gradually dieting (208) were the most commonly used WL methods among CS athletes, and their mean scores ranked first and second and both exceeded 200. This was expected because compared to other methods, the negative impact of these two methods is relatively less severe, and the implementation requirements (such as equipment, drugs, time) are fewer. Research showed that gradual WL before competition does not affect the generation of wrestlers’ torque and also helps to reduce body fat and increase the percentage of lean body mass [75]. Research on training practice always showed that equipment and time are important factors affecting practice [76,77,78]. Increasing the amount of exercise is a method of active sweating, which is also consistent with the daily training activities of athletes [78]. Compared with other methods, it has many advantages (e.g., improving skill proficiency and improving physical function). Nevertheless, there is also the worry that increasing the amount of exercise before competition may have some impact on the athlete’s pre-competition state (such as additional external load and psychological/mental fatigue) and increase the risk of injury [79,80,81]. A large number of studies have shown that coaches usually reduce the training volume before competition to regulate the athlete’s pre-competition state [76,77,78,82,83]. In contrast, gradual dieting is a good way to prevent the impact of additional exercise. There were six methods with a score of 101–200; these methods can be ranked from high to low as follows: restricting fluid ingestion (174), skipping one or two meals (152), training with rubber/plastic layers (150), intentionally training in heated training rooms (149), using saunas (134), and fasting (not eating all day) (125). It can be found that these methods are more extreme versions of gradual dieting and increasing exercise, meaning that they are all ways designed to help one lose weight by increasing sweat and limiting intake. There were six methods with a score of less than 100; these methods can be ranked from high to low as follows: spitting (96), the use of rubber/plastic layers without exercising (72), laxatives (46), diuretics (46), diet pills (40), and vomiting (26). It can be found that the greater the radicalness and implementation requirements of the method, the lower the mean score, especially in the case of the last four methods. Although these four methods are harmful, almost all studies showed that athletes have used or are still using these methods, and two studies even had a higher average score for these methods (129–181, 138–171), which is a dangerous signal. In fact, athletes usually use a variety of methods to lose weight, especially those with a greater magnitude of WL. However, until now, studies on the impact of WL methods have usually only explored one method, so the impact of using multiple methods on athletes is not clear.

### 4.4. Person Who Influenced Their WL Practice

It has been shown that athletes lack knowledge of WL [84]. Most of their WL practices are affected by someone’s direct instructions and the environment that has been formed around them. Studies have shown that coaches and trainers often encourage athletes to lose weight before competitions [47,84]. One study reported that 90% of coaches supervise athletes’ WL process and give athletes suggestions on WL methods [47]. For the environment that has been formed, one combat athlete interviewed in the study by Pettersson et al. [63] said, “(…) if I wouldn’t cut weight and everyone else does it, then I would be in trouble”. This evidence supports the view that a person with a specific role and environment is important. This review found that the mean influence score for coaches (368) was the highest, followed by training partners/fellows (321), physical trainers (281), other athletes (same sport) (278), former athletes (271), nutritionists/dietitians (236), parents (229), and doctors/physicians (192). This distribution is understandable because coaches are often responsible for almost everything related to the training and competition of athletes, and the mutual support, companionship, and influence between athletes is almost ubiquitous [85,86,87]. Interestingly and worryingly, qualified practitioners (doctors/physicians, nutritionists/dietitians), who can really provide scientific advice regarding WL, have the least influence on CS athletes. Only two studies showed that the scores of doctors/physicians and/or nutritionists/dietitians were more than 300 [6,54]. Unfortunately, the authors did not discuss this special phenomenon. A potential vicious cycle exists in the WL practice of CS, that is, unscientific WL practice is continuously transmitted from coaches and athletes to young athletes, while scientific WL advice stands outside such individuals’ scope of practice. In order to avoid this vicious cycle, doctors/physicians and nutritionists/dietitians should influence the practice of athletes by influencing coaches. Relevant sporting governing bodies and managers should improve the WL education of coaches to ensure that athletes can train and compete in a safe environment.

## 5. Conclusions

In summary, we found that WL is highly prevalent in CS athletes. The WL pattern of most CS athletes is similar, that is, they begin to lose weight two to three times a year after starting to participate in competitions and usually start doing so within 7–14 days before a competition to lose less than 5% of their BW. The most common methods used by CS athletes are increased exercise and gradual dieting, but the used methods also include potentially harmful methods (e.g., diet pills, vomiting), although these are less frequently used. Although the habitual practice of CS athletes may be relatively harmless, in some special cases, athletes also perform extreme WL practices (such as losing >10% of BW by multiple harmful methods within 7 days). What is particularly worrying is that scientific practitioners have little influence on their WL practices, which may form a vicious cycle of non-qualified influence. Therefore, it is urgent to find a way to break this cycle according to the current environment. Specifically, it is recommended to make rational use of the vast influence of coaches and athletes, educate them on WL, and find ways to enhance the influence of nutritionists to avoid unsafe WL practices.

## 6. Practical Application

Coaches should be aware of their influence on athletes’ WL practices and give full consideration to their influence to intervene in athletes’ WL practices. Specifically, coaches should (1) control the age at which athletes begin to lose weight; (2) plan the annual times, magnitude (kg and %BW), and time span of WL for athletes; (3) monitor the WL methods used by athletes and provide suggestions; and (4) improve their communication with nutritionists and doctors to obtain the necessary WL-related knowledge. Nutritionists and doctors should also improve communication with coaches, provide necessary education to coaches, and try to spread scientific knowledge about WL practices through the vast influence of coaches. Relevant managers should try to enact stricter pre-competition WL-related rules for the three sports of Sambo, MMA, and Muay Thai to reduce the harm caused by extreme WL practices to athletes.

## Figures and Tables

**Table 1:** The general information of the participants and prevalence of weight loss within the included studies.

Reference	Guilherme Giannini Artioli et al. [20]	Nikos Malliaropoulos et al. [43]	Rafael Kons et al. [42]	Ben-El Berkovich et al. [34]	Rafael L Kons et al. [3]	Nikola Todorović et al. [44]	Flavia Figlioli et al. [39]	Patrik Drid et al. [15]	Mathew Hillier et al. [40]	Rubens B. Santos-Junior et al. [13]	Marcelo Romanovitch Ribas et al. [51]	Leonardo Vidal Andreato et al. [52]	John Connor et al. [38]	Tyler White et al. [45]	Marijana Ranisavljev et al. [4]	Boris Dugonjić et al. [16]	Jonatas Ferreira Da Silva Santos et al. [19]	B.B. Vasconcelos et al. [7]	Marcelo Romanovitch Ribas et al. [53]	Stefano Amatori et al. [18]	Cecilia Castor-Praga et al. [36]	Léna Pélissier et al. [6]	Whye Lian Cheah et al. [37]	Hakan Yarar et al. [46]	Ng Qi Xiong et al. [54]	Reid Reale et al. [12]	Mean
Year																											
	2010	2017	2017	2016	2020	2021	2021	2021	2019	2019	2017	2014	2019	2021	2022	2019	2016	2023	2019	2020	2021	2022	2019	2020	2017	2017	
Country																											
	Brazil	England	Brazil	Israel	Brazil	12 countries	35 countries	20 countries	—	Brazil	Brazil	Brazil	Ireland	England	16 countries	8 countries	Brazil	Brazil	Brazil	Italy	Mexico	France	Malaysia	—	Malaysia	Australia	—
Sport																											
	Judo	Judo	Judo	Judo	Visually impaired judo	Sambo	Sambo	Sambo	MMA	MMA	MMA	MMA	MMA	Jiu-jitsu	Wrestling	Kickboxing	Taekwondo	Sanda	Muay Thai	Boxing	Wrestling, Taekwondo	Multiple combat sports	Multiple combat sports	Multiple combat sports	Multiple combat sports	Multiple combat sports	—
Sample size (*n*)																											
	822	255	12	108	30	47	103	199	314	179	25	8	30	115	120	61	116	71	20	164	160	168	65	502	40	260	—
Male (%)																											
	74	74	100	100	67	0	100	66	91	92	100	—	100	94	68	100	62	72	100	88	60	48	68	74	65	65	—
Female (%)																											
	26	26	0	0	33	100	0	34	9	8	0	—	0	6	32	0	38	28	0	12	40	52	32	26	35	35	—
Competitive level																											
	R, S, N, I	C, R, N, I	—	R, N, I	N, I	I	I	I	—	S, N, I	S, N	R, N	—	R, N, I	I	I	R, S, N, I	N	S, N, I	—	—	L, R N, I	Non-I	—	I	N, I	—
Regional (%)																											
	17	—	—	—	—	0	0	0	—	0	—	—	—	—	0	0	—	—	0	—	—	12	—	—	0	0	—
State (%)																											
	38	—	—	—	—	0	0	0	—	29	—	—	—	—	0	0	—	—	50	—	—	0	—	—	0	16	—
National (%)																											
	24	—	—	—	—	0	0	0	—	43	—	—	—	—	0	0	—	100	40	—	—	38	—	—	0	27	—
International (%)																											
	21	—	—	—	—	100	100	100	—	28	—	—	—	—	100	100	—	—	10	18	—	47	—	—	100	47	—
Age (year)																											
	19.3	28.1	23.3	14.6	26.2	23.1	24.2	22.7	27.3	25.0	24.4	22.0	25.5	29.3	27.6	24.2	21.5	22.5	27.7	22.9	13.3	24.0	22.9	20.9	21.0	23.2	23.3
Body weight (kg)																											
	70.0	76.6	87.2	58.0	70.7	62.7	75.5	71.6	80.2	77.0	79.5	74.3	78.3	—	76.2	73.9	64.6	65.1	80.1	68.9	48.9	67.6	—	—	62.2	69.5	71.2
Height (m)																											
	1.71	1.73	1.72	1.65	1.66	1.63	1.74	1.71	—	1.76	1.76	1.77	1.79	—	1.72	1.79	1.72	1.70	1.74	1.75	1.57	1.69	—	—	—	1.73	1.72
Number of competitions in past 12 month (*n*)																											
	—	—	—	—	—	—	—	—	—	2.0	3.0	—	2.5	—	—	4.8	6.8	—	4.5	—	—	—	—	—	—	7.1	4.4
Age at start of training (year)																											
	9.1	—	10.2	5.5	15.6	—	11.2	11.3	—	19	16	18.6	—	25.8	—	14.5	12.8	16	18.7	16.2	6.8	10.7	14.7	—	10.7	—	13.9
Age at start of competing (year)																											
	10.7	—	—	6.8	18.1	—	12.7	—	—	20	19.6	19.7	—	26.7	14.7	15.3	13.4	17.4	22.4	18.2	8.0	12.8	16.6	—	12.8	—	17.9
Off-season weight (kg)																											
	71.1	—	—	—	—	—	—	—	—	79	82.2	—	78.3	80.5	—	75.6	65.3	68.8	83.4	—	—	—	—	—	63.1	—	74.7
Prevalence of weight loss (%)																											
	86	84	100	80	67	89	90	87	97	100	100	100	97	59	83	100	81	90	100	88	96	95	66	100	93	88	89

Note: R: regional; S: state level; N: national level; I: international level; C: club level; L: local; Non-I: non-international level.

**Table 2:** Assessment of risk of bias using the Downs and Black tool.

Study	Guilherme Giannini Artioli et al. [20]	Nikos Malliaropoulos et al. [43]	Rafael Kons et al. [42]	Ben-El Berkovich et al. [34]	Rafael L Kons et al. [3]	Nikola Todorović et al. [44]	Flavia Figlioli et al. [39]	Patrik Drid et al. [15]	Mathew Hillier et al. [40]	Rubens B. Santos-Junior et al. [13]	Marcelo Romanovitch Ribas et al. [51]	Leonardo Vidal Andreato et al. [52]	John Connor et al. [38]	Tyler White et al. [45]	Marijana Ranisavljev et al. [4]	Boris Dugonjić et al. [16]	Jonatas Ferreira Da Silva Santos et al. [19]	B.B. Vasconcelos et al. [7]	Marcelo Romanovitch Ribas et al. [53]	Stefano Amatori et al. [18]	Cecilia Castor-Praga et al. [36]	Léna Pélissier et al. [6]	Whye Lian Cheah et al. [37]	Hakan Yarar et al. [46]	Ng Qi Xiong et al. [54]	Reid Reale et al. [12]
Q1	Y	Y	Y	Y	Y	Y	Y	Y	Y	Y	Y	Y	Y	Y	Y	Y	Y	Y	Y	Y	Y	Y	Y	Y	Y	Y
Q2	Y	Y	Y	Y	Y	Y	Y	Y	Y	Y	Y	Y	Y	Y	Y	Y	Y	Y	Y	Y	Y	Y	Y	Y	Y	Y
Q3	N	N	N	N	N	N	N	N	N	N	N	N	N	N	N	N	N	N	N	N	N	N	Y	N	N	N
Q4	Y	Y	Y	Y	N	Y	Y	Y	N	Y	Y	N	Y	Y	Y	Y	N	Y	N	Y	Y	Y	Y	D	Y	Y
Q5	Y	Y	D	D	D	D	D	D	D	D	Y	D	D	Y	Y	Y	D	Y	D	D	D	D	Y	D	D	D
Q6	Y	Y	Y	Y	Y	Y	Y	Y	Y	D	Y	D	Y	Y	Y	Y	D	Y	Y	Y	Y	Y	Y	D	Y	Y
Q7	N	N	N	N	N	N	N	N	N	N	N	N	N	N	N	N	N	N	N	N	N	N	Y	N	N	N
Q8	Y	Y	Y	Y	Y	Y	Y	Y	Y	Y	Y	Y	Y	Y	Y	Y	Y	Y	Y	Y	Y	Y	Y	Y	Y	Y
Q9	Y	Y	Y	Y	Y	Y	Y	Y	Y	Y	Y	Y	Y	Y	Y	Y	Y	Y	Y	Y	Y	Y	Y	Y	Y	Y
Q10	Y	Y	Y	Y	Y	Y	Y	Y	Y	Y	Y	Y	Y	Y	Y	Y	Y	Y	Y	Y	Y	Y	Y	N	Y	Y
Q11	Y	Y	Y	Y	Y	Y	Y	Y	Y	Y	Y	Y	Y	Y	Y	Y	Y	Y	Y	Y	Y	Y	Y	Y	Y	Y
Q12	N	Y	Y	Y	Y	Y	Y	Y	N	Y	Y	Y	Y	Y	Y	Y	Y	Y	Y	Y	Y	Y	Y	N	Y	Y
Q13	D	D	D	D	D	D	D	D	D	D	D	D	D	D	D	D	D	D	D	D	D	D	D	D	D	D
Q14	N	N	N	N	N	N	N	N	N	N	N	N	N	N	N	N	N	N	N	N	N	N	N	N	N	N
Q15	Y	Y	Y	Y	Y	Y	Y	Y	Y	Y	Y	Y	Y	Y	Y	Y	Y	Y	Y	Y	Y	Y	Y	Y	Y	Y
Q16	Y	Y	Y	Y	Y	Y	Y	Y	Y	Y	Y	Y	Y	Y	Y	Y	Y	Y	Y	Y	Y	Y	Y	Y	Y	Y
Q17	Y	Y	Y	Y	Y	Y	Y	Y	Y	Y	Y	Y	Y	Y	Y	Y	Y	Y	Y	Y	Y	Y	Y	Y	Y	Y
Q18	Y	Y	Y	Y	Y	Y	Y	Y	Y	N	N	N	Y	N	Y	Y	N	Y	N	Y	Y	Y	Y	N	Y	Y
Q19	Y	N	N	Y	Y	Y	Y	Y	Y	Y	N	N	Y	Y	Y	Y	Y	Y	N	Y	Y	Y	N	N	Y	Y
Q20	Y	Y	Y	Y	Y	Y	Y	Y	Y	Y	Y	Y	Y	Y	Y	Y	Y	Y	Y	Y	Y	Y	N	Y	Y	Y

Note. Y: yes; N: no; D: don’t know/comment. (1) Were the aims/objectives of the study clear? (2) Was the study design appropriate for the stated aim(s)? (3) Was the sample size justified? (4) Was the target/reference population clearly defined? (Is it clear who the research was about?) (5) Was the sample frame taken from an appropriate population base so that it closely represented the target/reference population under investigation? (6) Was the selection process likely to select subjects/participants that were representative of the target/reference population under investigation? (7) Were measures undertaken to address and categorize non-responders? (8) Were the risk factor and outcome variables measured appropriate to the aims of the study? (9) Were the risk factor and outcome variables measured correctly using instruments/measurements that had been trialed, piloted, or published previously? (10) Is it clear what was used to determined statistical significance and/or precision estimates? (e.g., p-values, confidence intervals) (11) Were the methods (including statistical methods) sufficiently described to enable them to be repeated? (12) Were the basic data adequately described? (13) Does the response rate raise concerns about non-response bias? (14) If appropriate, was information about non-responders described? (15) Were the results internally consistent? (16) Were the results presented for all the analyses described in the methods? (17) Were the authors’ discussions and conclusions justified by the results? (18) Were the limitations of the study discussed? (19) Were there any funding sources or conflicts of interest that may have affected the authors’ interpretation of the results? (20) Was ethical approval or participants’ consent attained?

**Table 3:** The weight history of combat sports athletes.

Reference	Guilherme Giannini Artioli et al. [20]	Nikos Malliaropoulos et al. [43]	Rafael Kons et al. [42]	Ben-El Berkovich et al. [34]	Rafael L Kons et al. [3]	Nikola Todorović et al. [44]	Flavia Figlioli et al. [39]	Patrik Drid et al. [15]	Mathew Hillier et al. [40]	Rubens B. Santos-Junior et al. [13]	Marcelo Romanovitch Ribas et al. [51]	Leonardo Vidal Andreato et al. [52]	John Connor et al. [38]	Tyler White et al. [45]	Marijana Ranisavljev et al. [4]	Boris Dugonjić et al. [16]	Jonatas Ferreira Da Silva Santos et al. [19]	B.B. Vasconcelos et al. [7]	Marcelo Romanovitch Ribas et al. [53]	Stefano Amatori et al. [18]	Cecilia Castor-Praga et al. [36]	Léna Pélissier et al. [6]	Whye Lian Cheah et al. [37]	Hakan Yarar et al. [46]	Ng Qi Xiong et al. [54]	Reid Reale et al. [12]	Mean
Sport																											
	Judo	Judo	Judo	Judo	Visually impaired judo	Sambo	Sambo	Sambo	MMA	MMA	MMA	MMA	MMA	Jiu-jitsu	Wrestling	Kickboxing	Taekwondo	Sanda	Muay Thai	Boxing	Wrestling, Taekwondo	Multiple combat sports	Multiple combat sports	Multiple combat sports	Multiple combat sports	Multiple combat sports	—
The number of people answered following questions																											
	688	214	12	86	20	47	103	173	314	179	25	8	29	68	120	61	94	64	20	164	153	160	43	502	37	229	139
Age at start of weight loss (y)																											
	12.6	—	—	12.5	20.6	14.9	16.0	15.8	—	21.0	—	19.0	—	—	19.2	18.5	17.7	18.8	—	18.4	—	17.1	—	14.7	15.9	18.3	17.1
Number of weight loss in past 12 months (*n*)																											
	3.0	—	—	2.8	—	—	3.2	—	—	2.0	3.3	2.0	—	—	—	3.2	3.0	—	3.9	1.3	—	—	—	2.6	2.2	4.7	2.9
Highest weight loss (kg)																											
	4.0	—	—	2.7	7.1	—	8.0	—	—	12.0	13.9	7.6	—	—	—	6.2	4.7	6.5	11.1	3.8	—	5.1	4.9	5.4	5.1	5.9	6.7
(and body weight %)																											
	6.0	—	—	4.7	10.0	—	10.6	—	—	15.6	17.5	10.2	—	—	—	8.4	7.3	10.3	13.9	5.5	—	7.5	—	—	8.0	8.5	9.6
Habitual weight loss (kg)																											
	1.6	—	3.5	1.5	2.8	3.3	5.0	5.3	7.1	10.0	9.3	5.0	6.2	—	3.6	3.5	1.9	2.8	8.5	1.3	—	2.8	3.4	—	2.9	2.8	4.3
(and body weight %)																											
	2.5	—	4.0	2.6	4.0	5.2	6.6	7.4	8.9	13.0	11.7	6.7	7.9	—	4.7	4.9	3.0	4.5	10.6	1.8	—	4.1	—	—	4.7	4.1	5.8
Typical body weight regain in a week after competition (kg)																											
	1.6	—	2.6	1.4	2.6	4.0	4.4	—	—	9.0	9.5	3.5	6.2	—	3.3	2.8	1.6	2.3	5.4	1.7	—	2.4	—	—	2.4	2.9	3.7
(and body weight %)																											
	2.3	—	3.0	2.4	3.7	6.3	5.8	—	—	11.7	11.9	4.4	7.9	—	4.3	3.8	2.5	3.5	6.7	2.5	—	3.5	—	—	3.9	4.2	5.0
Number of days over which weight is usually lost (days)																											
	7.0	—	—	8.0	16.0	11.9	11.7	11.9	—	—	24.5	12.0	—	—	10.6	9.6	11.4	19.3	29.1	10.1	—	13.1	—	—	11.9	12.8	13.6
0–7 days (%)																											
	72.1	—	33.3	—	—	—	—	—	—	—	—	—	—	50	—	—	—	—	—	—	—	—	37.2	36.9	—	—	45.9
8–14 days (%)																											
	14.8	—	33.3	—	—	—	—	—	—	—	—	—	—	31	—	—	—	—	—	—	—	—	25.6	32.6	—	—	27.5
15 days and over (%)																											
	13.1	—	33.3	—	—	—	—	—	—	—	—	—	—	19	—	—	—	—	—	—	—	—	24.6	30.5	—	—	24.1

**Table 4:** The weight loss methods used by combat sports athletes.

Reference	Guilherme Giannini Artioli et al. [20]	Nikos Malliaropoulos et al. [43]	Rafael Kons et al. [42]	Ben-El Berkovich et al. [34]	Rafael L Kons et al. [3]	Nikola Todorović et al. [44]	Flavia Figlioli et al. [39]	Patrik Drid et al. [15]	Mathew Hillier et al. [40]	Rubens B. Santos-Junior et al. [13]	Marcelo Romanovitch Ribas et al. [51]	Leonardo Vidal Andreato et al. [52]	John Connor et al. [38]	Tyler White et al. [45]	Marijana Ranisavljev et al. [4]	Boris Dugonjić et al. [16]	Jonatas Ferreira Da Silva Santos et al. [19]	B.B. Vasconcelos et al. [7]	Marcelo Romanovitch Ribas et al. [53]	Stefano Amatori et al. [18]	Cecilia Castor-Praga et al. [36]	Léna Pélissier et al. [6]	Whye Lian Cheah et al. [37]	Hakan Yarar et al. [46]	Ng Qi Xiong et al. [54]	Reid Reale et al. [12]	Mean
Sport																											
	Judo	Judo	Judo	Judo	Visually impaired judo	Sambo	Sambo	Sambo	MMA	MMA	MMA	MMA	MMA	Jiu-jitsu	Wrestling	Kickboxing	Taekwondo	Sanda	Muay Thai	Boxing	Wrestling, Taekwondo	Multiple combat sports	Multiple combat sports	Multiple combat sports	Multiple combat sports	Multiple combat sports	—
Increased exercise (more than usual)																											
	243	192	279	216	160	203	179	188	213	244	230	238	181	195	201	225	218	260	295	209	217	209	255	—	213	194	218
Gradual dieting to lose weight in 2 wk or more																											
	145	180	163	146	173	221	218	217	271	249	240	150	245	219	226	218	172	—	295	199	—	179	224	—	201	227	208
Restricting fluid ingestion																											
	144	151	213	95	143	183	157	167	201	237	268	163	233	129	141	164	155	192	295	115	183	153	113	—	149	201	174
Skipping 1 or 2 meals																											
	158	125	163	160	95	193	168	176	103	111	—	138	147	165	149	159	149	125	270	95	183	145	130	—	184	161	152
Training with rubber/plastic layers																											
	116	83	97	—	92	150	192	175	—	234	228	131	146	50	117	148	168	117	285	159	216	158	115	150	—	113	150
Training intentionally in heated training rooms																											
	154	90	187	86	113	157	171	168	117	184	214	138	114	94	151	126	154	173	295	120	178	119	150	—	149	114	149
Saunas																											
	56	100	121	35	87	184	198	196	160	176	248	113	159	120	180	166	79	63	210	75	187	120	57	115	138	152	134
Fasting (not eating all day)																											
	106	102	121	69	85	144	113	117	119	147	—	144	157	126	109	93	137	129	295	52	191	94	136	—	86	131	125
Spitting																											
	134	—	92	20	87	91	127	115	—	146	—	163	72	45	102	87	93	109	230	28	208	28	39	34	89	66	96
Use rubber/plastic layers without exercising																											
	90	45	71	—	95	89	114	110	—	—	—	63	7	41	80	41	45	—	—	55	161	86	69	—	—	37	72
Laxatives																											
	41	—	25	1	55	30	55	49	47	38	—	19	50	45	45	20	129	23	—	27	148	31	28	30	89	34	46
Diuretic																											
	32	—	33	1	58	38	40	38	55	70	—	88	19	23	31	16	150	27	—	19	138	27	27	20	81	22	46
Diet pills																											
	12	—	—	0	—	33	38	34	35	60	—	25	12	29	26	28	181	7	—	17	150	10	25	33	68	12	40
Vomiting																											
	11	—	17	6	48	23	27	30	6	9	—	—	0	32	27	5	—	6	—	10	171	16	36	—	36	11	26

**Table 5:** The influences of different roles on weight loss practice among combat sports athletes.

Reference	Guilherme Giannini Artioli et al. [20]	Nikos Malliaropoulos et al. [43]	Rafael Kons et al. [42]	Ben-El Berkovich et al. [34]	Rafael L Kons et al. [3]	Nikola Todorović et al. [44]	Flavia Figlioli et al. [39]	Patrik Drid et al. [15]	Mathew Hillier et al. [40]	Rubens B. Santos-Junior et al. [13]	Marcelo Romanovitch Ribas et al. [51]	Leonardo Vidal Andreato et al. [52]	John Connor et al. [38]	Tyler White et al. [45]	Marijana Ranisavljev et al. [4]	Boris Dugonjić et al. [16]	Jonatas Ferreira Da Silva Santos et al. [19]	B.B. Vasconcelos et al. [7]	Marcelo Romanovitch Ribas et al. [53]	Stefano Amatori et al. [18]	Cecilia Castor-Praga et al. [36]	Léna Pélissier et al. [6]	Whye Lian Cheah et al. [37]	Hakan Yarar et al. [46]	Ng Qi Xiong et al. [54]	Reid Reale et al. [12]	Mean
Sport																											
	Judo	Judo	Judo	Judo	Visually impaired judo	Sambo	Sambo	Sambo	MMA	MMA	MMA	MMA	MMA	Jiu-jitsu	Wrestling	Kickboxing	Taekwondo	Sanda	Muay Thai	Boxing	Wrestling, Taekwondo	Multiple combat sports	Multiple combat sports	Multiple combat sports	Multiple combat sports	Multiple combat sports	—
Coach																											
	296	—	375	—	410	347	332	345	—	—	440	—	372	—	317	402	—	350	495	—	365	268	360	—	433	353	368
Training partners/fellows																											
	—	—	241	—	340	257	255	254	—	—	446	—	396	—	260	—	—	—	415	—	219	327	384	—	378	—	321
Physical trainer																											
	211	—	175	—	395	279	234	243	—	—	—	—	259	—	221	256	—	340	395	—	210	357	405	—	335	174	281
Other athletes (same sport)																											
	284	—	225	—	295	219	258	241	—	—	448	—	—	—	249	269	—	—	—	—	—	—	—	—	—	290	278
Former athlete																											
	259	—	—	—	—	—	—	—	—	—	—	—	—	—	—	215	—	336	100	—	—	386	330	—	—	271	271
Nutritionist/Dietitian																											
	190	—	175	—	300	187	194	184	—	—	—	—	236	—	217	121	—	255	206	—	255	436	240	—	373	210	236
Parents																											
	228	—	183	—	270	270	233	248	—	—	180	—	130	—	214	195	—	246	0	—	303	412	282	—	308	191	229
Doctor/Physician																											
	151	—	142	—	250	174	192	183	—	—	228	—	163	—	180	128	—	186	100	—	151	447	219	—	213	149	192

## Data Availability

All data generated or analyzed during this study are included in this published article.
